# STAT5A induced LINC01198 promotes proliferation of glioma cells through stabilizing DGCR8

**DOI:** 10.18632/aging.102938

**Published:** 2020-04-04

**Authors:** Cheng Tan, Yimeng Dai, Xiaoyang Liu, Guifang Zhao, Weiyao Wang, Jia Li, Ling Qi

**Affiliations:** 1 Department of Neurology, China-Japan Union Hospital of Jilin University, Changchun 130033, Jilin, P.R. China; 2 Department of Radiology, China-Japan Union Hospital of Jilin University, Changchun 130033, Jilin, P.R. China; 3 The Sixth Affiliated Hospital of Guangzhou Medical University, Qingyuan People's Hospital, Qingyuan 511518, P.R. China; 4 Department of Pathophysiology, Jilin Medical University, Jilin 132013, P.R. China

**Keywords:** glioma, LINC001198, proliferation, DGCR8, STAT5A

## Abstract

Background: LINC01198 has been suggested to be able to predict overall prognosis for glioma; however, it has been little described in glioma.

Results: It was shown that LINC01198 was markedly enriched in neoplasmic tissues relative to normal controls; and that elevated LINC01198 significantly correlated with unfavorable overall prognosis. Moreover, activation of STAT5A, identified as transcription factor (TF), can induce the expression of LINC01198. DGCR8, a kind of RNA-binding proteins (RBPs), was identified to be able to bind with LINC01198 that can stabilize the DGCR8. Five differential miRNAs with most significant difference, including miR-21-5p, miR-34-5p, miR-1246, miR-4488 and miR-494, were obtainable after silencing of DGCR8.

Conclusions: Together, the data we presented here suggested that STAT5 induced LINC01198 promotes proliferation and motility of glioma cells through stabilizing DGCR8 in glioma cells.

Methods: Expression of LINC01198 was appraised by quantitative PCR (qPCR) and in situ hybridization (ISH) in glioma clinical specimens, totaling 100 cases. Post hoc statistical analysis was conducted. *In vitro*, LINC01198 was stably silenced or re-expressed by transfection with lentiviral-based vectors. Chromatin-immunoprecipitation (CHIP) was applied to identify the relevant TFs that can bind with LINC01198, which was corroborated with electrophoretic mobility shift (EMSA) assay. RNA-immunoprecipitation (RIP) was used to identify the RNA-binding protein that can bind with LINC01198. Moreover, miRNA microarray was used to screen out differential miRNAs after silencing of DGCR8.

## INTRODUCTION

Glioma, a malignant brain tumor that arises from the brain’s supportive tissue, is known as glial tumor [1]. The tumor is predominantly made up of abnormal astrocytic cells, but also contains a mix of different cell types (including blood vessels) and areas of dead cells. Glioma is aggressive that invades into regions of brain that are nearby. Exceedingly rare is for glioma to spread outside of the brain [2]. The molecular mechanism involved in the proliferation and invasion of glioma remains poorly understood in spite of an increasingly thorough depth of knowledge were acknowledged [3, 4].

Recent advances in whole-genome sequencing technology have led to the discovery of a new type of regulatory gene, that is long non-coding RNAs (hereafter referred to as lncRNAs), which are more than 200 bases in length and unable to translate [5, 6]. Increasing evidence emerged were suggestive of the critical roles lncRNAs played in cell proliferation, differentiation and some other biological processes, as comprehensively reviewed [5]. The dysregulation of lncRNAs has been discovered in various types of human diseases [7] including cancer [8, 9], with the molecular mechanism involved being diverse [10]. LINC001198, despite tentatively suggested to be able to predict overall prognosis of glioma [11], has been scarcely described in glioma. The biological roles LINC001198 played and its working mechanism implicated remains largely unknown that left to be further investigated.

In this report, we showed that LINC001198 was strikingly enriched in glioma tissues as compared with matched normal controls; and that up-regulated LINC001198 significantly correlated with inferior overall prognosis. *In vitro* glioma cells, LINC001198 was shown to be capable of promoting proliferation and invasion of glioma cells. Mechanistically, STAT5A was identified as a novel TF that can induce the expression of LINC001198. Moreover, DGCR8, a kind of RNA-binding proteins (RBPs), was found to be able to bind with LINC01198. In addition, LINC01198 was also discovered to be capable of stabilizing DGCR8. Five differential miRNAs with most significant difference, including miR-21-5p, miR-34-5p, miR-1246, miR-4488 and miR-494, were obtainable after silencing of DGCR8. Collectively, the data we showed here indicate that LINC01198 plays a pivotal role in the proliferation of glioma.

## RESULTS

### Up-regulated LINC01198 was linked with poor overall prognosis in glioma

**Figure 1:**
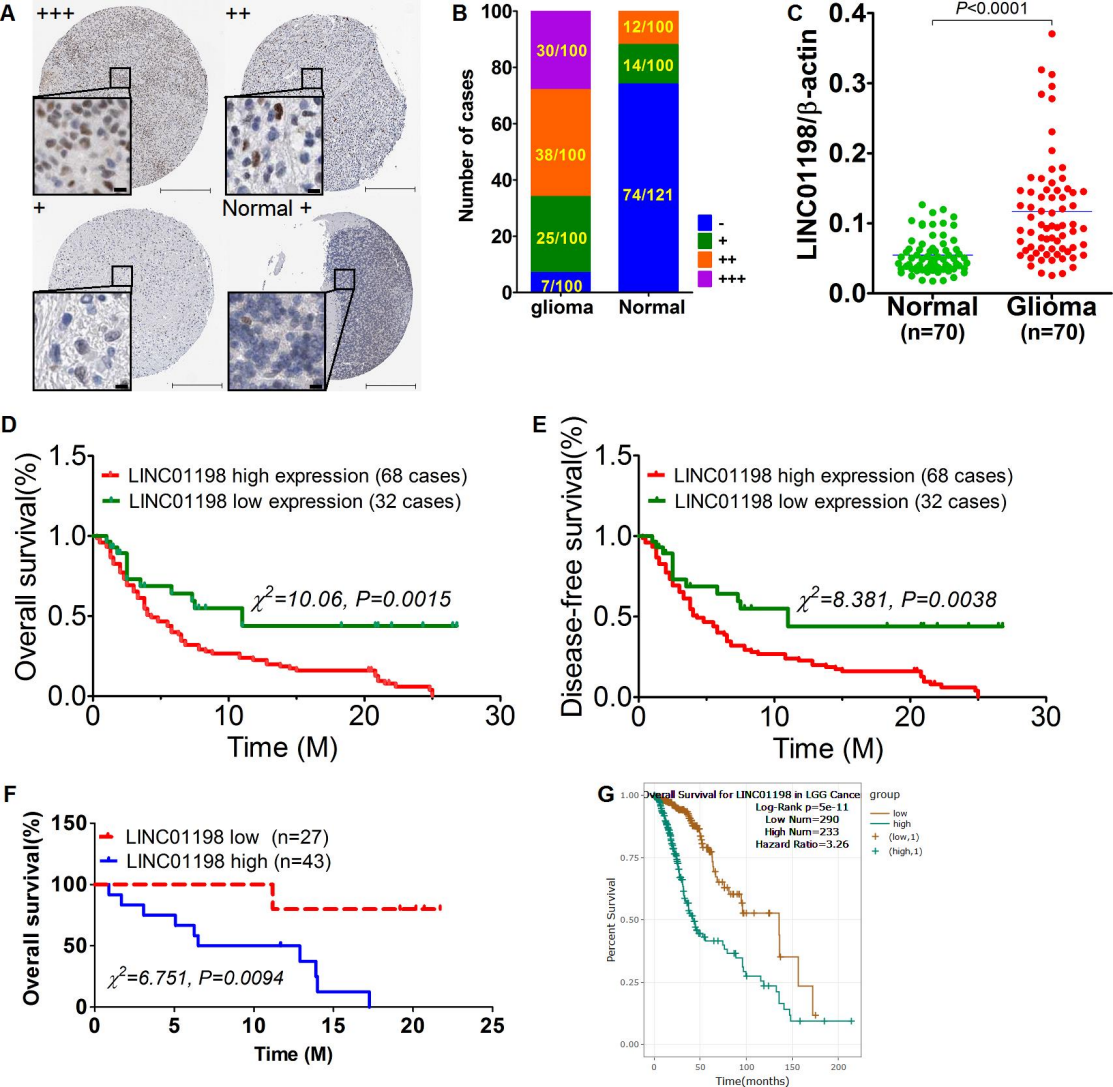
**Up-regulated LINC01198 was linked with poor overall prognosis in glioma.** (**A**) heterogeneous expression of LINC01198 in glioma as well as normal brain tissue, with LINC01198 level being markedly varied from strong positive, denoting as +++, to weak positive, denoting as + among different glioma cases, as evidenced by ISH staining. Scale bar, 50μm; scale bar in inset, 5μm. (**B**) the enumeration of LINC01198 expression evaluated by ISH in glioma and its paired normal controls, totaling 100. (**C**) qRT-PCR detection of LINC01198 in another independent cohort comprising 70 cases of fresh frozen glioma tissues and its matched normal controls. Two tailed, paired T-test was used to analyze the significant difference (t=8.220, df=69, P<0.0001). (**D**) Overall survival analysis of LINC01198 by Kaplan-Meier survival curve in glioma, totaling 100. Of which, the number of cases detected with high expression of LINC01198 by ISH was 68; the remainder was 32 with low expression of LINC01198. Log-Rank Chi-square was used to analyze the difference of survival (χ^2^=10.06, P=0.0015). (**E**) Likewise and incidentally, disease-free survival was also analyzed by Log-Rank Chi-square was used to analyze the difference of survival (χ^2^=8.381, P=0.0038). (**F**) Similarly, Overall survival analysis was performed of LINC01198 in 70 cases of fresh frozen glioma tissues, totaling 70 cases. Log-Rank Chi-square was used to analyze the difference of survival (χ^2^=6.751, P=0.0094). (**G**) Overall survival analysis of LINC01198 expression data in patients diagnosed with low grade glioma (LGG) derived from TCGA database.

The original report [11] came from glioma mentioning that LINC01198 can predict the prognosis. Suggested by the study, we attempted to appraise the expression level of LINC01198 by means of in situ hybridization (ISH) technique on glioma tissue microarray comprised 100 paired neoplastic tissues and its paired normal controls. Results of ISH showed that LINC01198 was strikingly enriched in glioma tissues relative to matched normal controls (Figure 1A and 1B). Given the potential limitations of ISH technique [12] that ISH results were subject to false positive, qRT-PCR was therefore picked up to confirm the expression of LINC01198 in another independent fresh frozen tissues, totaling 70 paired cases. Consistently, Results of qRT-PCR corroborated what was achieved by ISH method, exhibiting that LINC01198 was markedly elevated in glioma tissues in comparison with paired normal controls (Figure 1C). Subsequently, statistical and prognostic analyses were performed with Cross-Table and Kaplan-Meier survival analysis approaches, respectively. Kaplan-Meier survival analysis revealed that elevated LINC01198 was dramatically linked with inferior overall survival (Figure 1D) and disease-free survival (Figure 1E) in glioma. Meanwhile, the same held totally true for the survival analysis from additional 70 cases of fresh frozen glioma tissues (Figure 1F). To further confirm what we achieved regarding the prognostic significance of LINC01198 expression, analysis of the data regarding LINC01198 in glioma from TCGA dababase revealed that, up-regulation of LINC01198 was significantly correlated with the overall prognosis of glioma (Figure 1G). Cross-Table statistical analysis displayed that expression of LINC01198 was remarkably associated with tumor grade, and relapse (Table 1). No significant correlation can be identified between LINC01198 expression and other clinicopathological parameters comprising age, gender and histological subtype. Furthermore, multivariate COX regression analysis was performed exhibiting that LINC01198 expression was an independent prognostic factor in glioma (Table 2), in addition to tumor grade and relapse. Collectively, the data we gleaned here demonstrated Up-regulated LINC01198 was significantly linked with poor prognosis in glioma.

**Table 1:** Clinicopathological significance of LINC01198 expression in 100 cases of glioma tissues.

**Variable**	**group**	**Total**	**LINC01198 expression**		**χ^2^**	**P value**
			**Low (-, +)**	**High (++,+++)**		
Glioma		100	32	68	65.333	0.000
Paired normal control		100	88	12		
Age	<60	57	19	38	0.108	0.830
	≥60	43	13	30		
WHO grade						
	I+II	38	18	20	6.653	0.015
	III+IV	62	14	48		
Gender						
	female	47	17	30	0.709	0.520
	male	53	15	38		
Relapse						
	Yes	75	19	56	6.127	0.024
	No	25	13	12		
Histological subtype						
	Oligoastrocytic	39	11	28	3.105	0.216
	Astrocytic	35	15	20		
	Oligodendroglial	26	6	20

**Table 2:** Univariate and multivariate analysis of clinicopathological parameters and LINC01198 expression in glioma.

	**Univariate**				**Multivariate**		
	**HR**	**95%CI**	***P* value**		**HR**	**95%CI**	***P* value**
Gender	0.035	0.591 to 1.815	0.902		-0.222	0.443 to 1.448	0.462
Age (≥60 vs <60 year)	0.533	1.071 to 2.700	0.224		0.697	1.220 to 1.305	0.656
WHO grade	0.241	1.016 to 1.591	0.036		0.287	1.005 to 1.635	0.045
Relapse	1.132	1.246 to 7.710	0.015		0.254	1.005 to 1.845	0.039
LINC01198 expression	-0.375	0.516 to 0.914	0.010		-0.395	0.489 to 0.929	0.016

### LINC01198 enhanced proliferation of glioma cells

**Figure 2:**
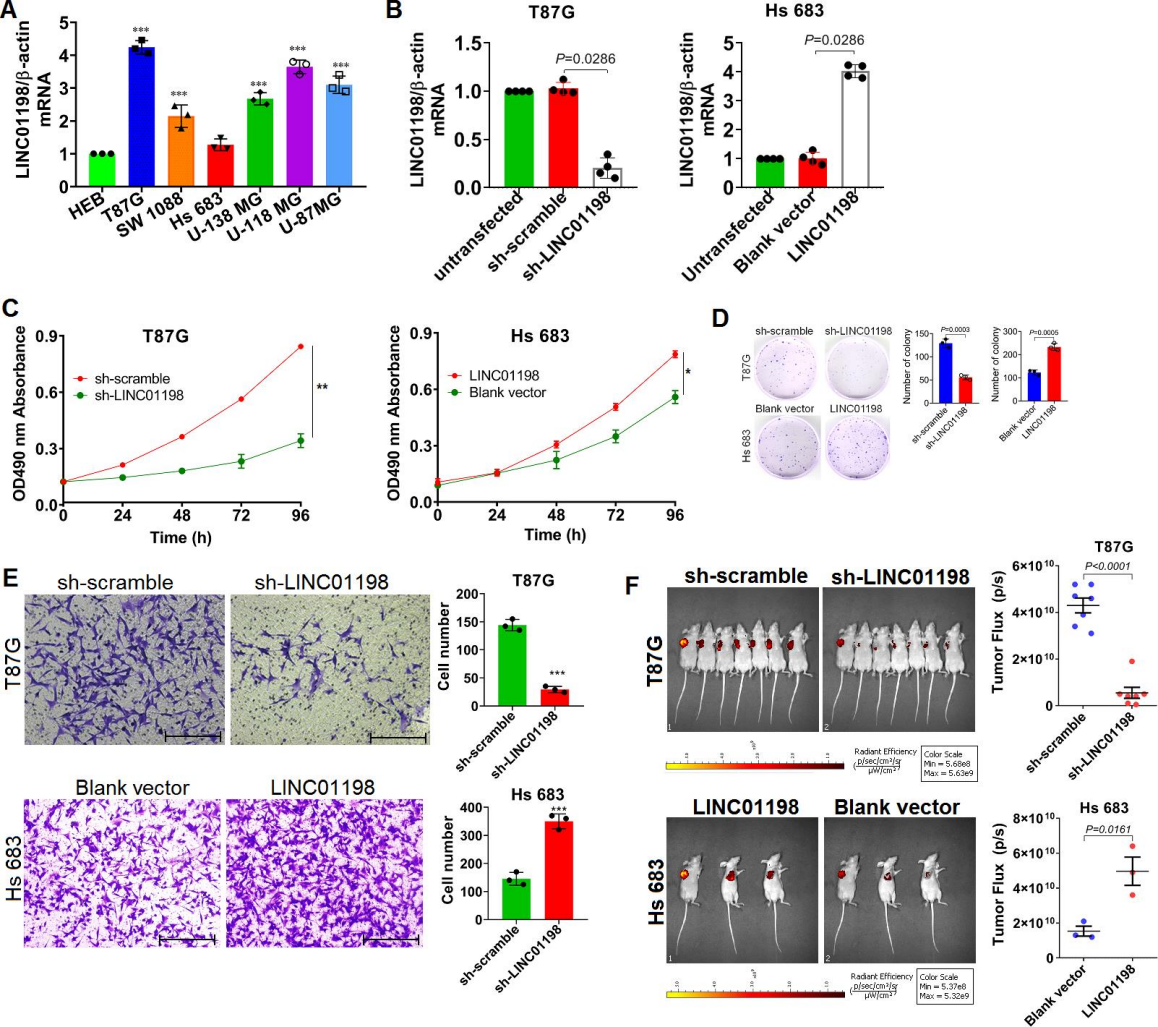
**LINC01198 enhanced proliferation and invasion of glioma cells.** (**A**) Basal level of LINC01198 was detected using qRT-PCR in the panel of glioma cell lines we enrolled and human normal glial cell line HEB, acted as normal control. qRT-PCR was actually performed independently three times (n=3). Shown was the stable result we achieved. Multiple comparisons were made using one way ANOVA analysis (Bonferroni approach). *** P<0.001 in comparison with HEB group. (**B**) Confirmation of LINC01198 variation by qRT-PCR after endogenous LINC01198 was being artificially manipulated using shRNA (T87G) or over-expression (Hs 683) strategy. The experiment was done independently four times; Shown was the stable result we achieved. Two-tailed, Mann-Whitney U test was employed to analyze the statistical difference (Mann-Whitney U=0, P=0.0286). (**C**) Proliferative variation of glioma cells was monitored by MTT approach after LINC01198 was being stably knocked down (T87G) or over-expressed (Hs 683). The experiment was carried out independently three times, shown was the representative one. Independent sample T-test was applied to analyze the proliferative difference, * P<0.05, ** P<0.01 compared with control group. (**D**) Clonogenesis assay was used to verify the clonogenic variations of glioma cells after LINC01198 was stably knocked down (T87G) or re-expressed (Hs 683). Two-tailed, unpaired T-test was used to analyze the colony formation difference (T87G, t=12.14, df=4, P=0.0003; Hs 683, t=10.14, df=4, P=0.0005); (**E**) Transwell assay was applied to analyze the invasive variation of glioma cells after LINC01198 was being stably knocked down (T87G) or over-expressed (Hs 683). Two-tailed, unpaired T-test was used to analyze the statistical difference (T87G, t=17.20, df=4, P<0.001; Hs 683, t=11.12, df=4, P<0.001). *** P<0.001 relative to control group. (**F**) Subcutaneous xenograft nude mice model was used to verify the proliferative variation of glioma cells whose LINC01198 was being stably knocked down (T87G) or over-expressed (Hs 683). Two tailed, unpaired T-test was used to analyze the significant difference (T87G, t=9.456, df=12, P<0.0001; Hs 683, t=4.003, df=4, P=0.0161).

To understand the biological roles of LINC01198 involved in the proliferation and invasion of glioma cells, Lentiviral-based short hairpin RNA (shRNA) interference vector and over-expression vector were constructed. A panel of six different kinds of glioma cell lines was enrolled and wherein the basal level of LINC01198 was evaluated using qRT-PCR. QRT-PCR detection revealed that among the six different kinds of glioma cell lines, T87G whose LINC01198 level was highest of all whereas the level of LINC01198 in Hs 683 was lowest (Figure 2A). Next to qRT-PCR detection, T87G cells were transfected with Lentiviral-shRNA-LINC01198 (hereafter referred to as sh-LINC01198), while Hs 683 cells were transfected with Lentiviral-LINC01198 (hereafter referred to as LINC01198). The negative controls that correspond to each were named sh-scramble and blank vector, respectively. Firstly, the knock down and over-expression efficiency were appraised using qRT-PCR, displaying that the lentiviral vectors can effectively work (Figure 2B). Based on which, MTT assay was performed to evaluate the proliferative variation after LINC01198 was stably knocked down or up-regulated. Results of MTT showed that LINC01198 can intensify the proliferation of glioma cells (Figure 2C), which was corroborated by clonogenesis assay (Figure 2D). Incidentally, Transwell assay was conducted to assess the invasive variation of glioma cells after the changing of LINC01198 expression. Data from Transwell assay exhibited that LINC01198 can profoundly promote the invasive abilities of glioma cells tested (Figure 2E). To further confirm what we observed *in vitro* cell lines, subcutaneous xenograft mouse model was employed to further evaluate the effect on proliferation LINC01198 exerted *in vivo*. Consistently, it exhibited that subcutaneous tumor volume was remarkably larger than that in control group (Figure 2F). Together, the data we achieved here demonstrated that LINC01198 promotes proliferation of glioma cells both *in vitro* and *in vivo*.

### STAT5A identified as transcription factor that regulates the transcription of LINC01198

**Figure 3:**
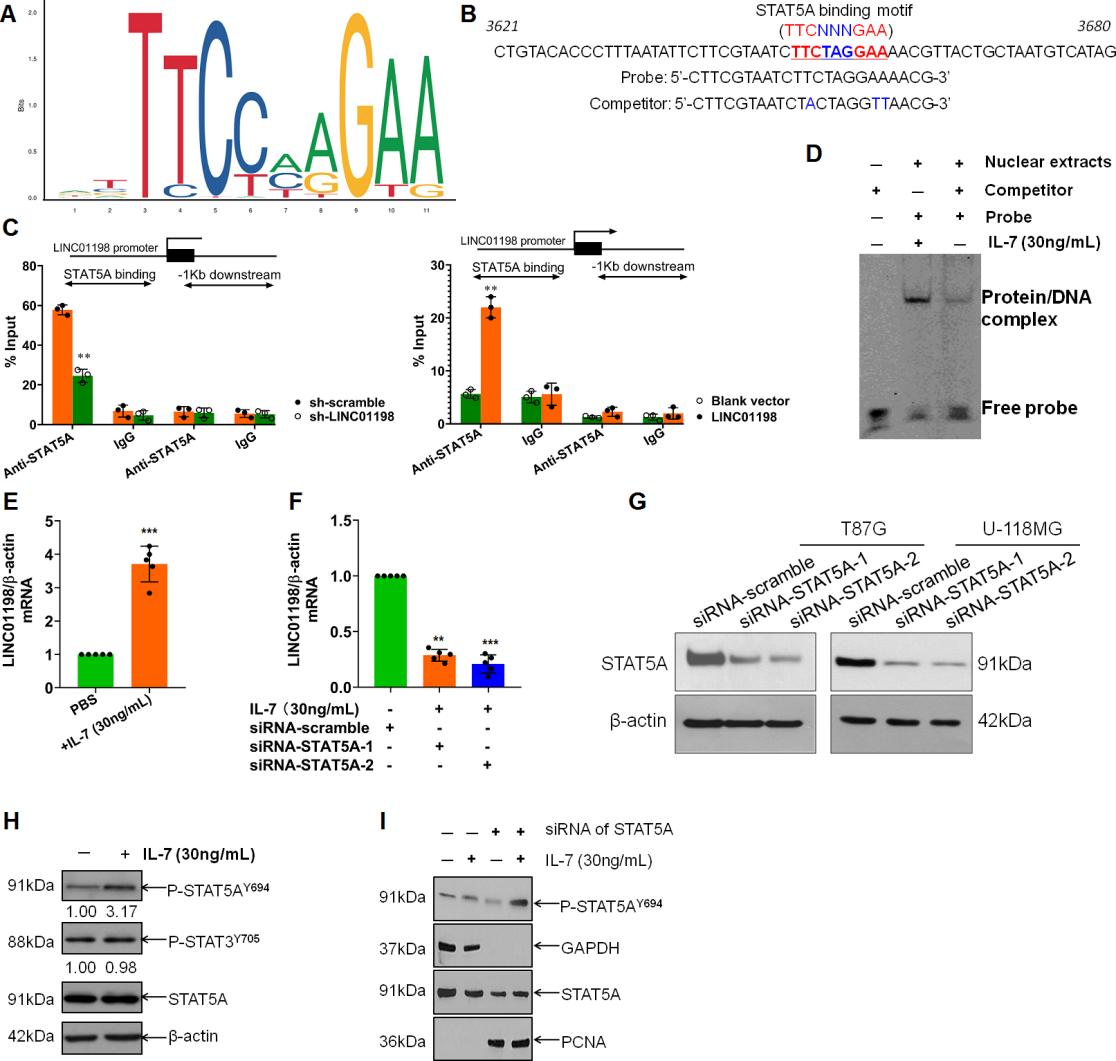
**STAT5A identified as transcription factor that regulates the transcription of LINC01198.** (**A**) Binding motif of STAT5A (TTCNNNGAA, here N denotes any nucleotide) in the promoter of LINC01198, predicted by JASPAR (http://jaspar.genereg.net/). (**B**) Representative binding position of STAT5A in the promoter of LINC01198 and the specific sequence of probe and its competitor probe involved in EMSA. (**C**) CHIP-qPCR analysis of transcriptional regulation of STAT5A on the promoter of LINC01198. Anti-STAT5A, CHIP-grade primary antibody to STAT5A; IgG, the isotype control of the primary antibody to STAT5A. The experiment was performed independently three times with triplicate in each time. Shown was the representative result. Two tailed, independent sample T-test was used to analyze the significant difference. ** P<0.01 compared with control. (**D**) EMSA analysis of the binding ability of STAT5A with the promoter of LINC01198. Here, competitor was the contraction of competitor probe. (**E**) Indirect regulation of STAT5A exerted by IL-7 (30ng/mL) over the expression of LINC01198 on mRNA level, as detected by qRT-PCR. (**F**) Similarly, indirect regulation of STAT5A exerted by IL-7 over the expression of LINC01198 on mRNA level in the presence of siRNA to STAT5A, as detected by qRT-PCR. Two-tailed, independent sample T-test was used to analyze the significant difference. ** P<0.01, *** P<0.001 in comparison with control group. (**G**) immunoblotting detection of the knock-down efficiency of siRNA to STAT5A in T87G and U-118MG glioma cells, shown were the representative figures picked out of candidates we collected. (**H**) STAT5A can be specifically and appreciably activated at its 694 Tyrosine in the presence of IL-7 whereas STAT3 can hardly be phosphorylated. (**I**) activated STAT5A was capable of translocation from cytoplasm to nucleus in the presence of IL-7, as exemplified by western-blot. Of note, PCNA used as internal loading control for nuclear protein. All the experiments related to western-blot were performed independently three times and presented were the representative ones singled out from candidates.

Having learned about the oncogenic traits of LINC01198, we next sought to get insight into the LINC01198. The promoter sequence of LINC01198 was subjected to bioinformatic analysis using JASPAR (http://jaspar.genereg.net/). Analysis revealed that there were STAT5A binding motifs in the promoter of LINC01198 (Figure 3A and 3B), meaning that STAT5A can be a putative TF for LINC01198. To verify the bioinformatic analysis JASPAR did, chromatin-immunoprecipitation (CHIP) was carried out to quantitatively evaluate the putative TF regulation of STAT5A on LINC01198. CHIP data showed that STAT5A can effectively operate as TF in the promoter of LINC01198, compared with negative control (Figure 3C). To further confirm, EMSA experiment was conducted exhibiting that STAT5A can acutely bind with the promoter probe of LINC01198, compared with control probe (Figure 3D). Subsequently, to further explore the regulation of STAT5A on the transcription of LINC01198, glioma cells were stimulated with recombinant human IL-7. qRT-PCR detection showed that LINC01198 was dramatically increased after stimulation with recombinant human IL-7, compared with control (Figure 3E); which can be abolished after STAT5A was silenced with specific siRNA (Figure 3F and 3G), strongly suggesting that activated STAT5A was required for up-regulation of LINC01198. In considering of STAT3, another important paralog of STAT5A; and that no specific commercial chemical inhibitor to STAT5A was available yet; to rule out the possible confounding influence that may be caused by STAT3, Immunoblot was performed to detect the variation of activated STAT3. It showed that IL-7 can specifically activate the activation of STAT5A, rather than STAT3 (Figure 3H). Next, Immunoblotting result revealed that, much like STAT3, nuclear translocation can occur to STAT5A once activated by IL-7 (Figure 3I). Overall, the data we showed here demonstrate that STAT5A was identified as transcription factor that can positively regulate the transcription of LINC01198.

### LINC01198 interacted with DGCR8 stabilizing DGCR8

**Figure 4:**
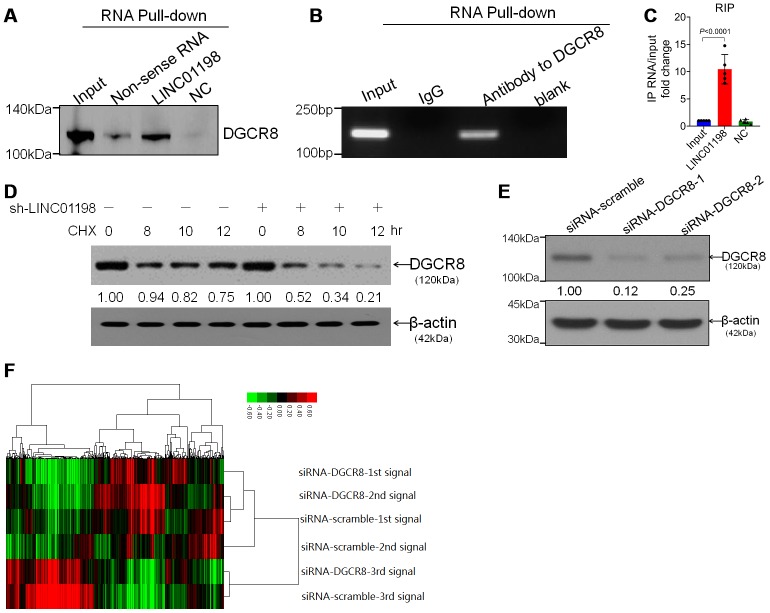
**LINC01198 interacted with DGCR8 stabilizing DGCR8.** (**A**) Immunoblotting detection of DGCR8 in the LINC01198 pull-down complex. LINC01198 fragment (hereafter referred to as LINC01198) was labeled with the biotin; Non-sense RNA was labeled with the biotin whose length was similar to that of LINC01198 fragment; NC was the LINC01198 without biotin label. The molecular weight of DGCR8 approximates 120 kilodalton (kDa), suggested by manufacturer’s instruction that accompanies. (**B**) The PCR product of RNA immunoprecipitation (RIP), run by agarose gel electrophoresis with 12% separating gel. (**C**) qRT-PCR verified that LINC01198 was accumulated in DGCR8-precipatated protein sample. Two-tailed, independent sample T-test was used to analyze the significant difference (t=7.794, df=8, P<0.0001). (**D**) immunoblotting detection of DGCR8 stability in the presence of Cycloheximide (CHX) at 12.5μg/mL in T87G cells transfected with or without sh-LINC01198. gray density was quantitated by Image J (NIH, Bethesda, MA, USA). (**E**) Knock down efficiency of siRNA to DGCR8 at two different interfering sites, as evaluated by western-blot. Shown were the representative ones singled out from candidates out of three independent experiments. (**F**) Cluster analysis of heat map of miRNA microarray (SurePrint, homo, Angilent, USA) performed on T87G and Hs 684 glioma cells transfected with siRNA to DGCR8. Transfection with siRNA-scramble was set as control.

Subsequently, we set out to identify the possible proteins that are unidentified that can interact with LINC01198. RNA binding protein immunoprecipitation (RIP) assay coupled with mass spectrometry analysis were performed. It was found that, it is DGCR8 that can directly interact with LINC01198 (Figure 4A–4C) with the aid of RIP analysis. Moreover, LINC01198 was discovered to be able to stabilize the DGCR8 (Figure 4D), in addition to it can bind with DGCR8. Given the key role of DGCR8 mediated in the microprocess [13, 14] and biogenesis of miRNA [15] involved in carcinogenesis, we next wonder whether the miRNA profile could be mediated by DGCR8 in glioma. Therefore, on the basis of successful silencing of DGCR8 using siRNA technique (Figure 4E), miRNA microarray analysis was undertaken (Figure 4E). The data presented here showed that, LINC01198 not only can bind with DGCR8 but also can stabilize it, explicitly suggestive of the scaffold role implicated in biogenesis of miRNA.

### DGCR8 produces a pro-growth miRNA profile that promotes cell proliferation

**Figure 5:**
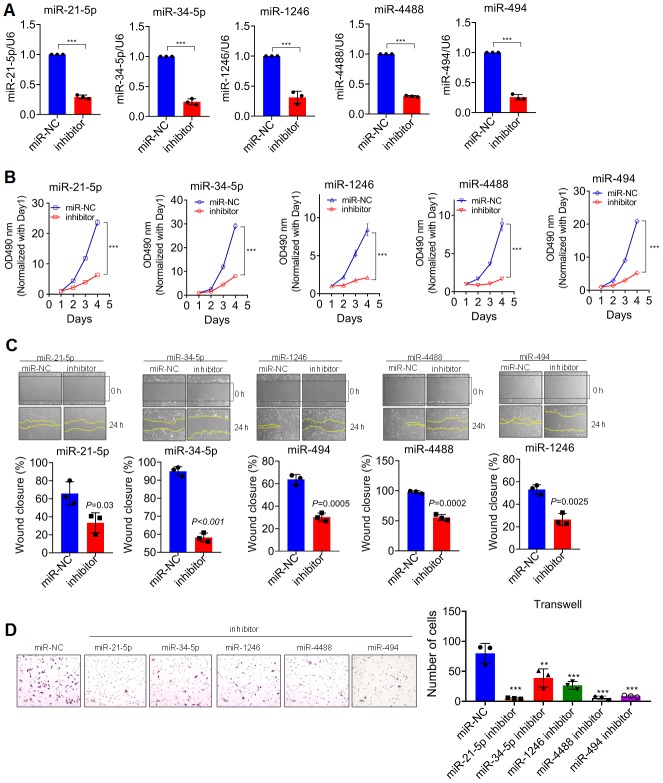
**DGCR8 produces a pro-growth miRNA Profile that promotes Cell Proliferation.** (**A**) knock-down efficiency detected by qRT-PCR of transient silencing of miRNA that down-regulated in T87G cells when DGCR8 was knocked down. miR-NC, miR-scramble; Inhibitor, a contraction of miRNA inhibitor. Two-tailed, unpaired T-test was used to analyze the significant difference (miR-21-5p, t=38.44, df=4, P<0.0001; miR-34-5p, t=26.19, df=4, P<0.0001; miR-1246, t=11.66, df=4, P<0.0001; miR-4488, t=16.9, df=4, P<0.0001; miR-494, t=28.33, df=4, P<0.0001). Here *** P<0.0001 compared with control. (**B**) MTT assay was performed on T87G cells whose endogenous miRNAs, as shown, were transiently knocked down. Two-tailed, unpaired T-test was used to analyze the significant difference, *** P<0.001 compared with control. (**C**) Wound closure assay was conducted in parallel with MTT assays to appraise the migratory variation in T87G cells. Two-tailed, independent T-test was used to analyze the significant difference. (**D**) Transwell assays were carried out to analyze the invasive variation after differential miRNAs were transiently knocked down in T87G cells. One way ANOVA (Bonferroni approach) was taken to analyze the significant difference among groups in comparison with control group transfected with miR-NC. Here, ** P<0.001, *** P<0.0001 relative to control.

In consideration that the expression of DGCR8 was tightly controlled in organism because of it is required for normal miRNA biogenesis and physiological functions; deregulations or alterations of DGCR8 expression linking with the aberrant expressions of miRNAs have therefore been detected in many diseases such as schizophrenia [16] and different kinds of cancers [14]. Inspired by these previous studies, we next sought to investigate the differential miRNA profile involved in the alteration of DGCR8 expression in glioma cells of our own. Firstly, DGCR8 expression was knocked down using specific siRNA with success, as evidenced by western-blot analyses (Figure 4E), followed by miRNA microarray was carried out (Figure 4F). Cluster heat map revealed that there were several miRNAs that were significantly down-regulated comprising miR-21-5p, miR-34-5p, miR-1246, miR-4488 and miR-494. Among these differential miRNAs that were screened out, GO analysis was conducted. The resultant analysis showed that these miRNAs that were markedly down-regulated with DGCR8 being significantly knocked down, were heavily implicated in cell proliferation (Figure 5B). To further confirm that these differential miRNA screened out were implicated in proliferation of glioma cells, all these five miRNAs were knocked down through transient transfection with miRNA inhibitor (Figure 5A). Then, MTT assay was performed. Results of MTT assay revealed that silencing of these five miRNAs was shown to be all able to significantly suppress the proliferation of glioma cells (Figure 5B). Meanwhile, both Transwell and Wound closure assays were also carried out to observe whether silencing of these miRNAs could have effects on invasion and migration. Data we collected showed that knock-down of them can markedly suppress the invasion and migration of glioma cells. Taken together, these data we obtained suggested that DGCR8 generated miRNA profile that can enhance the proliferation of glioma cells.

## DISCUSSION

In this report, LINC01198 was shown to be dramatically up-regulated in glioma tissues compared with matched normal controls. Striking elevation of LINC01198 was demonstrative of correlation with survival of glioma. In addition, biological roles of LINC01198 implicated in the proliferation and invasion of glioma cells were defined here, exhibiting that LINC01198 can profoundly enhance the proliferation and invasion of glioma cells. Moreover, STAT5A was identified as a transcriptional factor that can regulate the transpiration of LINC01198. Mechanistically, LINC01198 was discovered to be able to complex with DGCR8 thereby stabilizing DGCR8. These data we achieved here demonstrated that STAT5A induced LINC01198 promotes proliferation of glioma cells through triplex with DGCR8 and HIF-1α.

LINC01198, a contraction of Long Intergenic Non-Protein Coding RNA 1198, was initially captured in a glioma study [11] where LINC01198 was predicted to be remarkably associated with tumor grade and overall prognosis on clinical tissue level by virtue of bioinformatic data analysis, preliminarily implying the oncogenic nature of it. As a substantiation that follows, in a more recent study [17], LINC01198 was found to be up-regulated in glioma, and to be predictive of a poorer prognosis for patients with glioma. What is more, LINC01198 was shown to be capable of promoting the glioma cell proliferation and chemo-resistance. These previous data reported were highly consistent with the findings we presented here that elevated LINC01198 markedly correlated with tumor grade and unfavorable overall prognosis in glioma, strongly and explicitly indicative of the oncogenic trait of LINC01198 involved in the proliferation of glioma cells. Given that our study was just about glioma, the nature and expression level of LINC01198 in other types of tumors remains to be investigated in the experiment that follows. Therefore, the extrapolation of our data interpretation should be taken with caution. Despite the study emerged that was straightforwardly related with LINC01198 in glioma [17], the working mechanism, by which LINC01198 operates in the proliferation of glioma cells, has been little touched.

To get insight into the observation we made that LINC01198 was dramatically up-regulated in glioma compared with matched normal controls; attempt has been made naturally to subject the promoter sequence of LINC01198 to bioinformatically analyze with the aid of JASPAR software on line. The binding motif of STAT5A was identified in different sites of promoter sequence of LINC01198, which was further substantiated by CHIP-qPCR and EMSA analysis. The data we presented here demonstrate that STAT5A operates as a novel transcription factor for LINC01198 in glioma, which has never been reported before. Actually, several lines of evidence existed already revealing that STAT5A can be operative as direct transcription factor [18, 19] or indirect modulator [20, 21] for miRNA or mRNA STAT5A regulated in one way or another; In contrast, straightforward evidence has been less extensively studied showing that STAT5A works as transcription factor for lncRNA. Given this, our findings were novel in that STAT5A was identified as a TF that can regulate LINC01198 in glioma, which could account for the phenomena we described that LINC01198 was shown to be strikingly elevated in glioma relative to matched normal controls. Although STAT5A has not been touched in this study, including the clinicopathological significance of STAT5A expression and the possible biological functions implicated; there were several lines of evidence existed [22–24], explicitly and strongly supporting that activated STAT5A as TF heavily involved in the oncogenesis of glioma. Thus, there is no surprising that LINC01198 was observed to be drastically elevated in glioma. On the other, care also needs to be taken to interpret the finding we described here. We did not mean that STAT5A was the only TF for LINC01198. On the contrary, there was actually a great deal of other TFs that can be identified in the promoter of LINC01198, which might compensate or co-regulate with STAT5A which left to be further explored.

In exploring the regulatory motif of lncRNA, there was no getting away from the RNA-binding protein (RBP) [25]. LINC01198 was no exception here. Following the train of thought, we attempted to analyze and identify the potential RBPs that could bind with LINC01198. Eventually, DGCR8 was identified as a new RBP that can directly bind with LINC01198 that has not been reported ever before. Noticeably, DGCR8 protein stability was discovered to be modulated by LINC01198, strongly suggestive of the scaffold role of LINC01198. our observation was fundamentally in support of the recent finding by Chen WL et al [17] that the scaffold role LINC01198 operates in glioma. Despite the finding that DGCR8 was identified as RBP that can bind directly with LINC01198; here we were unable to clearly map the exact binding domain or amino acids of DGCR8 in this study, which therefore left to be localized in the experiment that follows. Additionally, given that expression of DGCR8 was tightly controlled in organism because it has been reported to be required for normal miRNA biogenesis and physiological functions [26], it stands to reason that deregulation of DGCR8 expression could be associated with the aberrant expression of miRNA. This notion, in effect, has been tested in some studies performed concerning DGCR8 in cancers [27–29]. Suggested by these earlier investigations, we tried to analyze and screen out the differential miRNAs using miRNA array platform that could be caused by artificial manipulation of DGCR8 expression. Data from miRNA array analysis revealed that some differential miRNAs that were implicated in pro-growth were obtainable. Importantly, some of the differential down-regulated miRNAs we screened out were fundamentally overlapping with the miRNAs screened by Ames HM and colleagues [30] in glial and glioneuronal tumors. However, our findings described here were totally different from observations made by Herbert KM and colleagues [31] in HeLa cells. The discrepancy between ours and study by Herbert KM et al may be explained by usage of different cell lines and tissue-specific expression of DGCR8. In our study, the biological roles as well as clinicopathological significance of DGCR8 expression in glioma remains unknown, which left to be investigated.

It has been well-established that miR-21 was extensively implicated in the carcinogenesis of different organs. In consideration of passenger strand and guide stand of miR-21 that has [32], miR-21-5p, as the guide strand of miR-21, has been little described in the context of glioma outside of one recent study [33], where there has been short of functional analysis surrounding miR-21-5p that was just mentioned as one of miRNAs that target genes coding for antioxidant mitochondrial enzymes. Herein, miR-21-5p was screened out as one of differential miRNAs after DGCR8 was knocked down; displaying that silencing of miR-21-5p can profoundly slow down the growth and motilities of T87G cells. Like miR-21-5p, miR-34-5p has been seldom reported in glioma except of one study [34] in spite of a large number of literatures abound regarding miR-34 in various types of cancer. The possible reason may be that previous investigators did not differentiate the guide strand or passenger strand of miRNAs they were interested in when studying on them. Our data revealed that miR-34-5p can promote both proliferation and motilities of T87G cells. Unlike miR-34-5p and miR-21-5p, there were several studies [30, 35, 36] available implying that stemness and invasion trait of miR-1246 in glioma, even can extend to other different types of cancer [37]. Here, we confirmed the oncogenic nature of miR-1246 in our own cases that miR-1246 accelerates the proliferation and invasion of glioma cells. It has been scarcely described surrounding miR-4488 in glioma except of the initial study [30] mentioning that miR-4488 was over-expressed in dysembryoplastic neuroepithelial tumors compared with the brain and other tumors related to brain. The data we presented here were indicative of the oncogenic trait of miR-4488 in glioma. In contrast, several lines of studies [38, 39] have suggested the straightforward relationship that miR-494 heavily mediated the proliferation and aggressiveness of glioma cells, which was corroborated by the evidence we presented here. Despite these findings, no direction regulation between LINC01198 and miR-129-5p was explored in our setting.

## CONCLUSIONS

In summary, the data we showed here suggested that STAT5 induced LINC01198 promotes proliferation and motility of glioma cells through stabilizing DGCR8 in glioma cells.

## MATERIALS AND METHODS

### Clinical specimen collection

Human glioma specimens were collected from the Department of Neurology, China-Japan Union Hospital of Jilin University. Approval of this study was granted by Medical Ethics Committee of Jilin University. Written informed consent was obtained from each patient enrolled. Adjacent normal brain tissue that corresponds to glioma tissues were taken from tissues that were 5cm away from tumor margin. The baseline characteristics of the patients were tabulated in Supplementary Table 1.

### Cell culture

Five kinds of glioma cell lines (T87G, SW1088, Hs 683, U-138MG, U-118MG, and U-87 MG), and one kind of normal human glial cell line named HEB were from the Institute of Biochemistry and Cell Biology the Chinese Academy of Sciences (Shanghai, China). Cells were cultured in RPMI 1640 or DMEM with 10% FBS (Gibco) and cultured at 37 with 5% CO_2_. The background information of these cell lines we enrolled were tabulated in Supplementary Table 2.

### MTT and clonogenic assays

Cells were plated onto 96-well culture plates and MTT (5 μg/mL) was added to each cells per day. MTT was removed after 4 h of incubation and then dimethyl sulphoxide was added to solubilize the formazan produced. Absorbency at 490 nm was monitored using BIO-RAD Model 680 Benchmark micro-plate reader (BIO-RAD, Hercules, CA, USA). For clonogenic assay, 100 cells were seeded on to 60 mm Petri dishes. After 2 weeks, cells were fixed and stained using 0.1% crystal viola (dissolved in methanol) for15 min, and the colonies were counted using Image J (NIH, Bethesda, MA, USA).

### RNA extraction and qRT-PCR analyses

Total RNA were extracted from glioma cancer tissues or glioma cell lines using TRIzol reagent (Invitrogen), following the manufacturers protocol. RNA was reverse transcribed to cDNA using a reverse transcription kit (Takara, Dalian, China). Real-time PCR was performed with SYBR Green (Takara, Dalian, China). GAPDH was used as reference for mRNA or lncRNA. Each sample was analyzed in triplicate. The primers were listed in Supplementary Table 3.

### RNA interference

Glioma cells were transfected with siRNA by using Lipofectamine 2000 (Invitrogen, Carlsbad, CA, USA), according to the manufacturer’s protocol. The cells were incubated for 48 h before use in assays. Cells were transfected with lentiviral based sh-LINC01198, sh-scramble, LINC01198 and blank vecor (multiplicity of infection, MOI=20) diluted by Enhanced Infection Solution (ENi.S, pH 7.4). Polybrane (10 μg/mL) was used to enhanced effect of infection. After 72 h transfection, cells were observed with green fluorescence, they were subjected to cell sorting by Flow Cytometer for further purity. The siRNA and shRNA sequences were listed in Supplementary Table 3.

### RNA immunoprecipitation (RIP)

RNA immunoprecipitation (RIP) experiments were performed by using a Magna RIP™ RNA-Binding Protein Immunoprecipitation Kit (Millipore, USA) according to the manufacturer’s instructions. Antibody for RIP assays of DGCR8 was from Abcam. RIP analysis was conducted in glioma cells using Magna RIP RNA-binding protein immunoprecipitation kit (Catalog number: 17-700, Millipore, Billerica, MA, USA) according to manufacturer’s instructions. Briefly, cells were collected after washing with cold PBS and RIP lysis buffer was added. The suspension was then centrifuged and 100 μL from each cell lysate was transferred to the RIP immunoprecipitation buffer, which contained Ago2-conjugated magnetic beads and IgG as a negative control (1:200, #3900, Cell Signaling Technology, Danvers, MA, USA). The magnetic beads were washed with RIP wash buffer and then incubated with proteinase K at 55 °C for 30 min. Subsequently, RNA was extracted for RT-qPCR analysis.

### RNA pull-down assay

Biotinylated RNAs were transcribed using Biotin RNA Labeling Mix (Roche, Swiss) and T7 RNA polymerase (Promega, Madison, WI, USA) and subsequently treated with RNase-free DNase I (Promega, Madison, WI, USA) and RNeasy Mini Kit (Catalog number: 74104, Qiagen, Hilden, German). Next, magnetic beads were added to each binding reaction sample and incubated at room temperature. Finally, the beads were washed, and eluted proteins were detected by western-blot and qRT-PCR analysis, respectively.

### Chromatin immunoprecipitation (ChIP) assay

ChIP assays were performed using CHIP KIT (Millipore, Billerica, MA, USA) following the manufacturer’s instruction. DGCR8 antibody was obtained from Cell Signaling (1: 50, # 6914, Cell Signaling Technology, Danvers, MA, USA). The ChIP primer sequences were listed in Supplementary Table 3. Quantification of immunoprecipitated DNA was performed using qPCR with SYBR Green Mix (Takara, Dalian, China). ChIP data was calculated as a percentage relative to the input DNA by the equation 2(Input Ct−Target Ct) ×0.1×100.

### Electrophoretic mobility shift (EMSA) assay

Nuclear extracts were first isolated from T87G cells and then nuclear proteins (5 μg) were mixed with biotin-labeled probes (Figure 2B, Supplementary Table 3) containing the STAT5A consensus sequence (50 fmol). These were then incubated at room temperature for 20 min. The protein-DNA mixtures were separated from unbound probe using a 5% polyacrylamide gel at 4 °C for 2 h in a Tris-glycine-EDTA running buffer. The gel was then transferred and detected using an ECL detection system (Sage Creation, Beijing, China).

### Immunoblotting

SDS-PAGE and western blots were performed following the standard protocols [31]. Antibody binding to bands was detected using an ECL detection system (Sage Creation). The primary antibodies used were specific to STAT5A (1:1000, #4807, Cell Signaling Technology, Danvers, MA, USA), DGCR8 (1:1000, #6914, Cell Signaling Technology), p-STAT5A^Y694^ (1:1000, #4322, Cell Signaling Technology), p-STAT3^Y705^ (1:1000, #9145, Cell Signaling Technology), PCNA (1:1000, #13110, Cell Signaling Technology), GAPDH (1:10000, ab181602, Abcam, Cambridge, UK).

### Wound closure and transwell assays

Cell migration was assessed by the wound closure assay. T87G cells were plated in 6-well plate at a concentration of 4×10^5^ cells per well and allowed to form a confluent monolayer for 24 h. Then, the monolayer was scratched with a sterile pipette tip (10 μL), washed with serum free medium to remove floating and detached cells, and photographed (time 0 h, 24 h) by inversion fluorescence microscope (Olympus, Takachiho Seisakusho, Japan). Cell culture inserts (24-well, pore size 8 μm; BD Biosciences) were seeded with 4×10^3^ cells in 100 μL of medium with 0.1% fetal bovine serum (FBS). Inserts pre-coated with Matrigel (40 μL, 1 mg/mL; BD Biosciences) were used for invasion assay. Media with 10% FBS (500 μL) was added to the lower chamber and served as a chemotactic agent. Noninvasive cells were wiped from the upper side of the membrane and cells on the lower side were fixed and stained with 0.1% crystal violet (dissolved in methanol) and counted using Image J software (NIH, Bethesda, MA, USA).

### Nude mice xenograft assay

Animal experiments were performed in accordance with the Care and use of Laboratory Animals with protocols approved by the Animal Care and Use Committee at Jilin University. Two kinds of glioma cell lines were used. T87G cells (6 ×10^6^ cells suspended in 0.1 mL sterile saline) transfected with lentiviral-based sh-LINC01198 (n=7) or sh-scramble (n=7), and Hs 683 cells transfected with lentiviral-based LINC01198 over-expression (n=3) or blank vectors (n=3) were injected subcutaneously into the axillary of five 6-week-old female BALB/c nude mice (Charles River Laboratory, Beijing). Four weeks later, all the mice were euthanatized and subjected to bioluminescence imaging for appraising the tumor size.

### In situ hybridization (HIS) assay

ISH analysis was carried out using a locked-nucleic acid (LNA) specific probe for LINC01198 (Exiqon, Vedbaek Denmark) on glioma tissue microarray comprised 100 paired dots. The sequence was 5’-CCGAAUGUCACAUGGGGUGUACUCC-3’ with 5’-DIG and 3’-DIG labeled.U6 snRNA was set as a positive control. Tissue array slide was treated with proteinase K (2 μg/mL). 3% H_2_O_2_ was used to block endogenous peroxidase activity. Hybridization was performed at 52°C overnight with 80 nM of DIG-labeled LNA probes. The staining intensity of LINC01198 was scored by two separate pathologists; scoring conflicts were resolved by consensus.

### Statistical analysis

The T-test or one-way analysis of variance (ANOVA) was applied to analyze the significant differences between groups. For analysis of the ISH immunoscore, significant difference was determined with Chi-square test and GraphPad Prism 8.0 version (GraphPad Software, Inc., La Jolla, CA, USA). Survival was analyzed using the Kaplan-Meier survival curve. All comparisons were two-tailed and when P score was less than 0.05 was considered significant.

## Supplementary Material

Supplementary Tables
