# Correction for: STAT5A induced LINC01198 promotes proliferation of glioma cells through stabilizing DGCR8

**DOI:** 10.18632/aging.203346

**Published:** 2021-07-15

**Authors:** Cheng Tan, Yimeng Dai, Xiaoyang Liu, Guifang Zhao, Weiyao Wang, Jia Li, Ling Qi

**Affiliations:** 1 Department of Neurology, China-Japan Union Hospital of Jilin University, Changchun 130033, Jilin, PR China; 2 Department of Radiology, China-Japan Union Hospital of Jilin University, Changchun 130033, Jilin, PR China; 3 The Sixth Affiliated Hospital of Guangzhou Medical University, Qingyuan People's Hospital, Qingyuan, 511518, PR China; 4 Department of Pathophysiology, Jilin Medical University, Jilin 132013, PR China

**Keywords:** correction

Original article: Aging. 2020; 12:5675–5692.  . https://doi.org/10.18632/aging.102938

**This article has been corrected: Figure 2F** has a new panel summarizing results from the T87G/sh-LINC01198 mouse group, which replaced the duplicated panel for the T87G/sh-scramble group. There are also new panels for the Hs 686 control group and the overexpressing LINC01198 group because of the accidental duplication of the original panels. The corrected **Figure 2F** was produced using the original data. These corrections do not change the content of the publication and do not affect the conclusion drawn from this research.

The corrected **Figure 2F** is given below.

**Figure 2:**
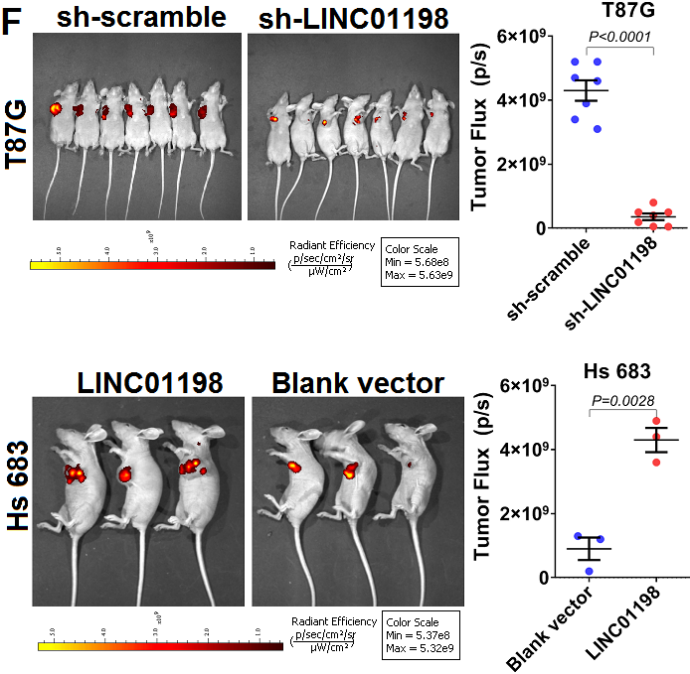
**LINC01198 enhanced proliferation and invasion of glioma cells.** (**F**) Subcutaneous xenograft nude mice model was used to verify the proliferative variation of glioma cells whose LINC01198 was being stably knocked down (T87G) or over-expressed (Hs 683). Two tailed, unpaired T-test was used to analyze the significant difference (T87G, t=9.456, df=12, P<0.0001; Hs 683, t=4.003, df=4, P=0.0161).

