# Supplementary material for: STAT5A induced LINC01198 promotes proliferation of glioma cells through stabilizing DGCR8
Source: Aging (Albany NY). 2020 Apr 4;12(7):5675–92. doi: 10.18632/aging.102938 (PMC7185146; doi:10.18632/aging.102938)
Supplement: Supplementary Tables [file aging-12-102938-s001..pdf]

SUPPLEMENTARY TABLES

Supplementary Table 1. The baseline characteristics of the patients involved.

Sample	Gender	Age	Subtype	Grade	Relapse	Survive	Time (D)
GLIOMA-1	Female	57	Astrocytic	GRADE IV	Yes	Dead	180
GLIOMA-2	Male	43	Astrocytic	GRADE II	No	Live	750
GLIOMA-3	Male	61	Astrocytic	GRADE IV	Yes	Dead	90
GLIOMA-4	Male	50	Astrocytic	GRADE III	No	Live	750
GLIOMA-5	Female	47	Astrocytic	GRADE III	No	Live	750
GLIOMA-6	Male	67	Oligoastrocytic	GRADE IV	Yes	Live	750
GLIOMA-7	Female	65	Astrocytic	GRADE IV	Yes	Dead	330
GLIOMA-8	Male	58	Oligoastrocytic	GRADE I	Yes	Dead	870
GLIOMA-9	Female	65	Astrocytic	GRADE III	Yes	Dead	870
GLIOMA-10	Male	53	Astrocytic	GRADE IV	Yes	Live	750
GLIOMA-11	Male	65	Oligoastrocytic	GRADE III	Yes	Dead	480
GLIOMA-12	Female	51	Astrocytic	GRADE III	No	Live	750
GLIOMA-13	Male	61	Oligoastrocytic	GRADE III	Yes	Live	750
GLIOMA-14	Female	46	Oligoastrocytic	GRADE IV	Yes	Dead	330
GLIOMA-15	Male	56	Astrocytic	GRADE II	No	Dead	330
GLIOMA-16	Female	62	Astrocytic	GRADE III	No	Dead	110
GLIOMA-17	Female	58	Oligoastrocytic	GRADE III	Yes	Live	750
GLIOMA-18	Male	53	Oligoastrocytic	GRADE III	No	Dead	870
GLIOMA-19	Female	48	Astrocytic	GRADE I	Yes	Live	750
GLIOMA-20	Female	36	Oligoastrocytic	GRADE II	Yes	Live	750
GLIOMA-21	Female	55	Astrocytic	GRADE III	No	Dead	870
GLIOMA-22	Male	47	Oligoastrocytic	GRADE IV	No	Dead	150
GLIOMA-23	Female	59	Astrocytic	GRADE III	Yes	Live	750
GLIOMA-24	Male	60	Oligoastrocytic	GRADE III	Yes	Live	750
GLIOMA-25	Female	53	Astrocytic	GRADE IV	Yes	Live	750
GLIOMA-26	Male	52	Oligoastrocytic	GRADE II	No	Live	750
GLIOMA-27	Female	53	Astrocytic	GRADE III	No	Dead	570
GLIOMA-28	Male	58	Oligoastrocytic	GRADE III	Yes	Dead	330
GLIOMA-29	Female	61	Oligoastrocytic	GRADE I	Yes	Dead	270
GLIOMA-30	Male	54	Astrocytic	GRADE II	Yes	Live	750
GLIOMA-31	Male	50	Astrocytic	GRADE III	No	Live	750
GLIOMA-32	Female	68	Astrocytic	GRADE IV	Yes	Live	750
GLIOMA-33	Male	53	Astrocytic	GRADE II	Yes	Dead	750
GLIOMA-34	Male	68	NA	GRADE III	Yes	Dead	240
GLIOMA-35	Male	56	Oligoastrocytic	GRADE IV	Yes	Dead	660
GLIOMA-36	Female	55	Astrocytic	GRADE III	Yes	Dead	930
GLIOMA-37	Female	65	NA	GRADE I	Yes	Live	750
GLIOMA-38	Male	59	Oligodendroglial	GRADE IV	Yes	Dead	840
GLIOMA-39	Female	49	Astrocytic	GRADE III	Yes	Dead	300
GLIOMA-40	Male	39	Astrocytic	GRADE III	Yes	Dead	150
GLIOMA-41	Female	49	Oligodendroglial	GRADE III	Yes	Dead	750
GLIOMA-42	Male	56	Oligoastrocytic	GRADE II	Yes	Dead	750
GLIOMA-43	Female	52	Oligodendroglial	GRADE I	Yes	Live	750
GLIOMA-44	Male	60	Oligodendroglial	GRADE I	Yes	Live	750
GLIOMA-45	Female	54	Oligoastrocytic	GRADE I	Yes	Dead	750
GLIOMA-46	Male	64	Oligodendroglial	T4N2M0	Yes	Dead	360
GLIOMA-47	Female	52	Oligoastrocytic	GRADE II	Yes	Live	750
GLIOMA-48	Male	69	Oligodendroglial	GRADE IV	Yes	Live	750
GLIOMA-49	Female	53	Oligoastrocytic	GRADE IV	Yes	Dead	870
GLIOMA-50	Male	62	Oligodendroglial	GRADE I	Yes	Dead	840
GLIOMA-51	Female	56	Astrocytic	GRADE III	Yes	Dead	90
GLIOMA-52	Male	63	Oligoastrocytic	GRADE III	Yes	Live	750
GLIOMA-53	Female	50	Oligodendroglial	GRADE III	Yes	Live	750
GLIOMA-54	Male	67	Oligoastrocytic	GRADE I	Yes	Dead	60
GLIOMA-55	Female	68	Oligodendroglial	GRADE II	Yes	Live	750

GLIOMA-56	Male	47	Oligodendroglial	GRADE II	Yes	Dead	750
GLIOMA-57	Female	59	Oligoastrocytic	GRADE II	Yes	Dead	750
GLIOMA-58	Male	48	Oligoastrocytic	T3N1M1	Yes	Dead	720
GLIOMA-59	Female	57	Oligoastrocytic	GRADE I	Yes	Dead	510
GLIOMA-60	Male	65	Oligodendroglial	GRADE III	Yes	Dead	600
GLIOMA-61	Female	66	Oligodendroglial	GRADE III	Yes	Dead	930
GLIOMA-62	Male	63	Astrocytic	GRADE III	Yes	Dead	750
GLIOMA-63	Male	56	Oligoastrocytic	GRADE I	Yes	Dead	750
GLIOMA-64	Female	65	Oligodendroglial	GRADE III	Yes	Dead	750
GLIOMA-65	Female	58	Oligoastrocytic	GRADE IV	Yes	Dead	750
GLIOMA-66	Female	50	Astrocytic	GRADE III	Yes	Dead	360
GLIOMA-67	Male	69	Oligodendroglial	GRADE IV	Yes	Dead	300
GLIOMA-68	Female	77	Oligoastrocytic	GRADE I	Yes	Dead	750
GLIOMA-69	Male	68	Oligodendroglial	GRADE III	Yes	Dead	750
GLIOMA-70	Female	58	Astrocytic	GRADE II	Yes	Dead	780
GLIOMA-71	Female	67	Oligoastrocytic	GRADE I	Yes	Dead	750
GLIOMA-72	Male	69	Oligoastrocytic	GRADE III	Yes	Dead	750
GLIOMA-73	Male	57	Oligoastrocytic	GRADE I	Yes	Dead	750
GLIOMA-74	Male	66	Oligodendroglial	GRADE I	Yes	Dead	600
GLIOMA-75	Male	50	Astrocytic	GRADE IV	Yes	Dead	660
GLIOMA-76	Female	69	Oligoastrocytic	GRADE IV	Yes	Dead	450
GLIOMA-77	Female	50	Oligodendroglial	GRADE IV	Yes	Live	750
GLIOMA-78	Male	64	Oligoastrocytic	GRADE IV	Yes	Dead	750
GLIOMA-79	Female	53	Oligodendroglial	GRADE I	Yes	Dead	750
GLIOMA-80	Male	67		GRADE I	Yes	Dead	750
GLIOMA-81	Male	67	Oligodendroglial	GRADE II	Yes	Dead	750
GLIOMA-82	Female	69	Oligoastrocytic	GRADE II	Yes	Live	750
GLIOMA-83	Male	48	Oligoastrocytic	GRADE I	Yes	Dead	750
GLIOMA-84	Female	64	Astrocytic	GRADE II	Yes	Live	750
GLIOMA-85	Male	55	Oligoastrocytic	GRADE III	Yes	Live	750
GLIOMA-86	Male	67	Oligodendroglial	GRADE IV	Yes	Live	750
GLIOMA-87	Female	66	Oligoastrocytic	GRADE IV	Yes	Dead	210
GLIOMA-88	Male	64	Oligodendroglial	GRADE II	Yes	Dead	750
GLIOMA-89	Male	66	Oligoastrocytic	GRADE IV	Yes	Dead	750
GLIOMA-90	Male	62	Oligodendroglial	GRADE I	Yes	Dead	690
GLIOMA-91	Female	68	Astrocytic	GRADE IV	Yes	Dead	690
GLIOMA-92	Male	49	Astrocytic	GRADE IV	Yes	Dead	720
GLIOMA-93	Female	59	Oligodendroglial	GRADE IV	Yes	Dead	750
GLIOMA-94	Male	50	Oligoastrocytic	GRADE II	Yes	Dead	750
GLIOMA-95	Male	68	Oligodendroglial	GRADE III	Yes	Dead	810
GLIOMA-96	Female	56	Astrocytic	GRADE IV	Yes	Dead	150
GLIOMA-97	Female	53	Astrocytic	GRADE IV	Yes	Dead	750
GLIOMA-98	Female	43	Oligodendroglial	GRADE I	Yes	Live	750
GLIOMA-99	Female	60	Oligoastrocytic	GRADE IV	Yes	Dead	600
GLIOMA-100	Male	66	Oligodendroglial	GRADE II	Yes	Dead	240

Supplementary Table 2. The background information of these cell lines we enrolled.

ATCC No™	Name	Species	Source	Disease
CRL-1688 ™	T87G	human	Brain	glioblastoma,multiforme
HTB-12™	SW 1088	human	Brain	astrocytoma
HTB-138™	Hs 683	human	Brain	glioma
HTB-16™	U-138MG	human	Brain	glioblastoma
HTB-15™	U-118MG	human	Brain	glioblastoma, astrocytoma
HTB-14™	U-87MG	human	Brain	glioblastoma, astrocytoma
	HEB	human	Brain	normal glial

Supplementary Table 3. The sequences of primers used in qRT-PCR and CHIP analysis.

Gene name	Sense (5'-3')	
hsa-miR-21-5p-RT	CTCAACTGGTGTCGTGGAGTCGGCAATTCAGTTGAGTCAACA	
hsa-miR-21-5p-F	ACACTCCAGCTGGGTAGCTTATCAGACTGA	
hsa- miR-21-5p-R	TGGTGTCGTGGAGTCG	
hsa-miR-34a-5p-RT	CTCAACTGGTGTCGTGGAGTCGGCAATTCAGTTGAGACAACC	
hsa-miR-34a-5p-F	ACACTCCAGCTGGGTGGCAGTGTCTTAGCT	
hsa- miR-34a-5p-R	TGGTGTCGTGGAGTCG	
hsa-miR-1246-RT	CTCAACTGGTGTCGTGGAGTCGGCAATTCAGTTGAGCCTGCT	
hsa-miR-1246-F	ACACTCCAGCTGGGAATGGATTTTTGGAG	
hsa- miR-1246-R	TGGTGTCGTGGAGTCG	
hsa-miR-4488-RT	CTCAACTGGTGTCGTGGAGTCGGCAATTCAGTTGAGCGCCGG	
hsa-miR-4488-F	ACACTCCAGCTGGGAGGGGGCGGGCTCC	
hsa- miR-4488-R	TGGTGTCGTGGAGTCG	
hsa-miR-494-5p-RT	CTCAACTGGTGTCGTGGAGTCGGCAATTCAGTTGAGAGAGAAGA	
hsa-miR-494-5p-F	ACACTCCAGCTGGGAGGTTGTCCGTGTTGTC	
hsa- miR-494-5p-R	TGGTGTCGTGGAGTCG	
U6-F	GTGCTCGCTTCGGCAGCACATATACTAAAATTGGAA	
U6-R	ATCCAGTGCAGGGTCCGAGG	
hsa-U6-RT	GTCGTATCCAGTGCAGGGTCCGAGGTATTCGCACTGGATACGACAAAATA	
LINC01198-F	CCTTTCTCGGGGAAGATGAC	
LINC01198-R	GCAACTGCTGGACGATAACAA	
	**Sense (5'-3')**	**antisense (5'-3')**
hsa-miR-21-5p mimics	UAGCUUAUCAGACUGAUGUUGA	AACAUCAGUCUGAUAAGCUAUU
hsa-miR-34a-5p mimics	UGGCAGUGUCUUAGCUGGUUGU	AACCAGCUAAGACACUGCCAUU
hsa-miR-1246 mimics	AAUGGAUUUUUGGAGCAGG	UGCUCCAAAAAUCCAUUUU
hsa-miR-4488 mimics	AGGGGGCGGGCUCCGGCG	CCGGAGCCCGCCCCCUUU
hsa-miR-494-5p mimics	AGGUUGUCCGUGUUGUCUUCUCU	AGAAGACAACACGGACAACCUUU
hsa-miR-21-5p inhibitor	UCAACAUCAGUCUGAUAAGCUA	
hsa-miR-34a-5p inhibitor	ACAACCAGCUAAGACACUGCCA	
hsa-miR-1246 inhibitor	CCUGCUCCAAAAAUCCAUU	
hsa-miR-4488 inhibitor	CGCCGGAGCCCGCCCCCU	
hsa-miR-494-5p inhibitor	AGAGAAGACAACACGGACAACCU	
